# Do *DSM-5* Eating Disorder Criteria Overpathologize Normative Eating Patterns among Individuals with Obesity?

**DOI:** 10.1155/2014/320803

**Published:** 2014-06-26

**Authors:** Jennifer J. Thomas, Katherine A. Koh, Kamryn T. Eddy, Andrea S. Hartmann, Helen B. Murray, Mark J. Gorman, Stephanie Sogg, Anne E. Becker

**Affiliations:** ^1^Eating Disorders Clinical and Research Program, Massachusetts General Hospital, Boston, MA 02114, USA; ^2^Department of Psychiatry, Harvard Medical School, Boston, MA 02114, USA; ^3^Institute for Psychology, University of Osnabrück, 49074 Osnabrück, Germany; ^4^Massachusetts General Hospital Weight Center, Boston, MA 02114, USA; ^5^Department of Global Health & Social Medicine, Harvard Medical School, Boston, MA 02115, USA

## Abstract

*Background. DSM-5* revisions have been criticized in the popular press for overpathologizing normative eating patterns—particularly among individuals with obesity. To evaluate the evidence for this and other *DSM-5* critiques, we compared the point prevalence and interrater reliability of *DSM-IV* versus *DSM-5* eating disorders (EDs) among adults seeking weight-loss treatment. *Method.* Clinicians (*n* = 2) assigned *DSM-IV* and *DSM-5* ED diagnoses to 100 participants via routine clinical interview. Research assessors (*n* = 3) independently conferred ED diagnoses via Structured Clinical Interview for *DSM-IV* and a *DSM-5* checklist. *Results*. Research assessors diagnosed a similar proportion of participants with EDs under *DSM-IV* (29%) versus *DSM-5* (32%). *DSM-5* research diagnoses included binge eating disorder (9%), bulimia nervosa (2%), subthreshold binge eating disorder (5%), subthreshold bulimia nervosa (2%), purging disorder (1%), night eating syndrome (6%), and other (7%). Interrater reliability between clinicians and research assessors was “substantial” for both *DSM-IV* (*κ* = 0.64, 84% agreement) and *DSM-5* (*κ* = 0.63, 83% agreement). *Conclusion*. *DSM-5* ED criteria can be reliably applied in an obesity treatment setting and appear to yield an overall ED point prevalence comparable to *DSM-IV*.

## 1. Introduction

In 2013,* DSM-5* reorganized its eating disorder classification system with the objective of enhancing clinical utility. Changes included the revision of diagnostic criteria and merging of feeding and eating disorders into a single chapter [1]. Of particular relevance to individuals with obesity,* DSM-5* elevated binge eating disorder (BED) from a provisional research diagnosis to a formal diagnostic category.* DSM-5* also introduced the new term, avoidant/restrictive food intake disorder (ARFID), to replace and extend* DSM-IV* feeding disorder of infancy and early childhood. Lastly, the residual category (EDNOS) was reconfigured and renamed other specified feeding or eating disorder (OSFED)—implemented when a clinician specifies the reason that an individual does not meet full criteria for a specific feeding or eating disorder (e.g., subthreshold BN, subthreshold BED, and night eating syndrome (NES))—or unspecified feeding or eating disorder (UFED), when full diagnostic criteria are not met but the reason remains unspecified.

Eating disorders and obesity are highly comorbid, and this may be particularly true for presentations recently added to* DSM-5*. For example, the 12-month BED prevalence in the National Comorbidity Survey Replication (NCS-R) was just 1.2% among US adults, but 85% of those with BED were overweight or obese [2]. Similarly, NES has a point prevalence of 1% in the general population but affects 6–14% of individuals seeking treatment related to obesity [3]. Similarly, investigators have recently commented on the changing “weightscape” of BN, in which a growing number of patients with BN are overweight [4]. Moreover, of potential relevance to the clinical presentation of ARFID, a recent study found that 45% of adults who self-identified as “picky eaters” were overweight or obese [5].

It is important to note that the* DSM-5* Eating Disorders Work Group specifically decided* not* to make obesity a psychiatric diagnosis, stating that “genetic, physiological, behavioral, and environmental factors that vary across individuals contribute to the development of obesity; thus, obesity per se is not considered a mental disorder” (page 1238) [6]. It would therefore be unfortunate if* DSM-5* changes inadvertently pathologized normative eating behaviors among individuals with obesity, who already face discrimination in the workplace, health care facilities, educational institutions, the media, and interpersonal relationships [7]. Furthermore, classifying normative eating behavior as pathological could generate unnecessary referrals that would contribute to rising healthcare costs.

Since proposed* DSM-5* criteria were posted for public comment in 2010, the field has raised three primary critiques. The most vociferous came from* DSM-IV* forefather Allen Frances, who warned in his book* Saving Normal* that BED is a “fake mental disorder” (page 183) representing nothing more than “gluttony” (page 184) [8]. He and psychologist Thomas Widiger expressed concern that BED and other new diagnoses would “go from not currently recognized as mental disorders to become among the most common of the psychiatric disorders, potentially creating false epidemics of misidentified pseudopatients” (page 122) [9]. The claim that BED is a “fake” disorder was not supported in the World Health Organization's recent World Mental Health epidemiological study, which highlighted greater role impairment among individuals with BED compared to healthy controls [10]. However, more research is needed to determine the degree of impairment associated with* DSM-5* BED specifically. Furthermore, the concern that* DSM-5* revisions will cause an avalanche of new BED cases has not received empirical support. For example, in a secondary analysis of NCS-R data comparing 12-month BED prevalence under* DSM-IV* versus* DSM-5*, rates increased only from 1.6% to 1.7% in women and remained the same in men (0.8%) [11]. However, this study did not focus specifically on an overweight population and did not screen for ARFID and* DSM-5* OSFED examples, which may be especially prevalent among individuals with obesity.

The second critique was that* DSM-5* revisions may do little to reduce the large proportion of eating disorder cases previously relegated to the EDNOS (now OSFED) category [12]. Available data suggest that, while many individuals with* DSM-IV* EDNOS will be shifted into the major diagnostic categories, a substantial group will still fall into the residual category. For example, in one study of US females with lifetime eating disorders, the majority did not meet criteria for AN, BN, or BED and could only be diagnosed with residual eating disorders under both* DSM-IV* (67.9% EDNOS) and* DSM-5* (53.3% OSFED) [13]. However, no study has looked specifically at obese populations, where the prevalence of* DSM-5* OSFED remains unknown.

A third critique of* DSM-5* revisions was that, because* DSM-5* introduces new constructs, clinicians could have trouble applying these more complex criteria in routine practice [9]. This concern is reasonable given the history of a low reliability between clinical and research diagnoses for psychiatric disorders. Whereas diagnostic agreement between structured interviews and clinical diagnoses was substantial in a meta-analysis of* DSM-IV* eating disorders (*κ* = 0.70) [14] little is known about interrater reliability under* DSM-5*. A recent study found fair to substantial (*κ* = 0.54 to 0.80) test-retest reliability among researchers for* DSM-5* eating disorders [15], and another study found adequate (*κ* = 0.56) test-retest reliability for* DSM-5* BED among clinicians [16]. However, no study to date has examined the interrater reliability of* DSM-5* eating disorder diagnoses among clinicians versus research assessors, who may differ in their interpretation of diagnostic criteria [17].

Therefore, the purpose of this study was to evaluate the prevalence and reliability of* DSM-5* eating disorder diagnoses among individuals seeking weight-loss treatment. To our knowledge, this is the first study that features a comprehensive interview of* DSM-5* eating disorder constructs in an overweight/obese sample, rather than inferring* DSM-5* diagnoses from* DSM-IV-*based interviews. In contrast to* DSM-5* critiques, we hypothesized that (1) the point prevalence of eating disorders would be comparable under* DSM-IV* and* DSM-5*; (2) individuals with either a* DSM-IV* or* DSM-5* eating disorder diagnosis would have higher levels of psychopathology and impairment in comparison to healthy controls, supporting the convergent validity of* DSM-5* revisions; (3) significantly fewer participants would be diagnosed with* DSM-5* OSFED compared to* DSM-IV *EDNOS; and (4) interrater reliability between clinicians and research assessors would be similar under* DSM-IV* and* DSM-5*.

## 2. Methods

### 2.1. Participants

We recruited study participants from a hospital-based weight treatment center in the Northeastern US, which offers multidisciplinary management of overweight and obesity including nutrition counseling, behavioral weight loss, and bariatric surgery. We invited 147 patients consecutively evaluated by one of two participating psychologists from October 2011 to January 2012 to take part. (All patients referred to the weight management center receive a comprehensive psychiatric evaluation as part of routine care. The two participating psychologists conducted evaluations with approximately 80% of patients referred to the center during this four-month period, with the remainder of patients being evaluated by part-time clinicians or trainees who did not recruit participants for the research study). Of these, 68% (*N* = 100) took part. Demographic characteristics are presented in Table 1.

### 2.2. Diagnostic Assessment

After receiving approximately 30 minutes of training on the new criteria by study investigators, clinicians (*n* = 2 Ph.D. psychologists) diagnosed* DSM-IV* and* DSM-5* eating disorders via routine clinical interview. Research assessors (*n* = 3 Ph.D. psychologists) later independently conferred diagnoses via the Structured Clinical Interview for* DSM-IV *and a* DSM-5 *checklist [18] (see Section 2.3.1) during a telephone interview. The research interviews were audio recorded to allow for examination of interrater reliability among research assessors, which was evaluated in 20 randomly selected cases. Subsequently, the research assessor sent a link, via secure email, to an online survey of self-report assessments (see Section 2.3.2) presented though REDCap (an electronic data capturing system) [19]. The Partners Human Research Committee approved the study protocol.

### 2.3. Measures

We used the following measures to assess the point prevalence of* DSM-IV* and* DSM-5* eating disorders.

#### 2.3.1. Interview Measures

*Structured Clinical Interview for DSM-IV (SCID-IV), Eating Disorder Module.* The SCID-IV is a semistructured interview instrument that assesses* DSM-IV* Axis I disorders [20]. We used the eating disorders module only. Interrater reliability in the present study was high: *κ* = 0.87 (almost perfect according to Landis and Koch [21]) with 95% agreement (i.e., research assessors agreed on the specific eating disorder diagnosis or noncase status in 19 of the 20 cases randomly selected for double coding).

*Diagnostic Interview for DSM-5 Feeding and Eating Disorders.* This interview-based assessment was created by B. Timothy Walsh, Chair of the* DSM-5* Eating Disorders Work Group, to determine eating disorder presence according to* DSM-5* criteria [18]. Interrater reliability within the present study was high: *κ* = 0.87 (almost perfect according to Landis and Koch [21]) with 95% agreement (i.e., researcher assessors agreed on the specific eating disorder diagnosis or noncase status in 19 of 20 cases randomly selected for double coding).

#### 2.3.2. Self-Report Measures

We used the following questionnaires to compare eating pathology, general psychopathology, and clinical impairment among participants diagnosed with eating disorders versus those without eating disorders to establish the convergent validity of* DSM-5* categories.

*Eating Disorder Examination-Questionnaire.* The EDE-Q is the self-report version of a standard interview measure of eating disorder psychopathology [22]. The global score represents the severity of attitudinal pathology in four domains: restraint, eating concern, shape concern, and weight concern. Internal consistency of the EDE-Q global score in the present study was 0.89.

*Eating Disorder Inventory-3.* The EDI-3 assesses eating pathology and general psychological constructs of potential etiological relevance to eating disorders [23]. Internal consistency of the EDI-3 eating disorder risk subscale (including drive for thinness, bulimia, and body dissatisfaction) in the present study was 0.94.

*Beck Depression Inventory-II (BDI-II).* The BDI-II measures depression severity with items that reflect* DSM-IV* criteria for major depressive disorder [24]. Internal consistency of the BDI-II in the present study was 0.93.

*Clinical Impairment Assessment (CIA).* The CIA measures functional impairment associated with eating disorder symptoms in three domains (personal, cognitive, and social) [25]. Internal consistency of the CIA in the present study was 0.95.

*Body Checking Questionnaire (BCQ).* The BCQ assesses the nature and frequency of body checking behaviors [26]. It measures checking related to overall appearance, specific body parts, and idiosyncratic checking. Internal consistency of the BCQ in the present study was 0.96.

*State-Trait Anxiety Inventory (STAI).* The STAI is a commonly used measure of general anxiety symptoms [27]. In the present study, we used only the 20 items assessing trait anxiety. Internal consistency of the STAI trait subscale in the present study was 0.93.

### 2.4. Statistical Analyses

We calculated the overall prevalence of* DSM-IV* BN and EDNOS (including BED) as well as* DSM-5* BN, BED, ARFID, and OSFED. (We did not include anorexia nervosa, pica, and rumination disorder in overall prevalence estimates because none of these disorders was a focus of clinical attention at the weight-loss clinic. We refer interested readers to a separate paper that details the frequency and characteristics of pica and rumination behavior in the current sample [28].) To evaluate the critique that applying* DSM-5* criteria would increase the overall prevalence of eating disorders, we compared eating disorder prevalence under* DSM-IV* versus* DSM-5* using McNemar's test for dependent proportions. To test our hypothesis that both* DSM-IV* and* DSM-5* eating disorder criteria would identify individuals with higher levels of psychopathology and impairment in comparison to healthy controls, thus supporting the convergent validity of* DSM-5* diagnoses, we compared scores on self-report measures of eating and general psychopathology between those who did and did not have an eating disorder using a series of independent sample *t*-tests. To correct for family-wise error, we used a Bonferroni correction, dividing the standard alpha level by six (the number of self-report measures), which provided a more conservative alpha level of 0.0083. To test our hypothesis that significantly fewer participants would receive a residual eating disorder diagnosis (i.e., EDNOS, OSFED) under* DSM-5* compared to* DSM-IV*, we used a McNemar's test for dependent proportions. Lastly, to evaluate interrater reliability of clinical versus research diagnoses under* DSM-IV* and* DSM-5*, we calculated Cohen's kappa and interpreted it according to Landis & Koch criteria (0.0–0.20 “poor,” 0.21–0.40 “fair,” 0.41–0.60 “moderate,” 0.61–0.80 “substantial,” and 0.81–1.00 “almost perfect”) [21].

## 3. Results

### 3.1. Eating Disorder Prevalence under DSM-IV versus DSM-5

Table 2 presents eating disorder research diagnoses under* DSM-IV* and* DSM-5*. We did not find a significant difference in overall eating disorder point prevalence (including both formal and residual categories) under* DSM-IV* versus* DSM-5* (*P* = ns). Applying* DSM-IV *criteria, 29 participants (29%) met criteria for an eating disorder, including 2% (*n* = 2) with BN, 9% (*n* = 9) with BED, and 18% (*n* = 18) with EDNOS. Applying* DSM-5 *criteria, 32 participants (32%) met criteria for an eating disorder, including 2% (*n* = 2) with BN, 9% (*n* = 9) with BED, and 21% (*n* = 21) with OSFED. No participants were diagnosed with ARFID.

### 3.2. Convergent Validity

Table 3 presents means and standard deviations of eating and general psychopathology among individuals diagnosed with either a* DSM-IV* or a* DSM-5* eating disorder. Regardless of whether* DSM-IV* or* DSM-5* criteria were applied, individuals diagnosed with an eating disorder had significantly higher scores (consistent with greater psychopathology, risk, or distress/impairment) than individuals not diagnosed with an eating disorder, on the EDE-Q, EDI-3 Eating Disorder Risk subscale and CIA (*P*'s < 0.0001) as well as the BCQ and STAI (*P*'s < 0.01). However, individuals with eating disorders had higher BDI-II scores only when* DSM-IV* (*P* = 0.005), but not* DSM-5* (*P* = 0.014), criteria were applied.

### 3.3. Prevalence of Residual Eating Disorders

Contrary to our hypothesis, the proportion of participants diagnosed with residual eating disorders did not differ significantly under* DSM-IV* versus* DSM-5* (*P* = ns). Applying* DSM-IV *criteria, EDNOS (including BED, which was listed as an EDNOS example in* DSM-IV*) accounted for 93% (*n* = 27) of eating disorders cases. Applying* DSM-5* criteria, OSFED accounted for 66% (*n* = 21) of cases. Of these, 29% (*n* = 6) had NES, 24% (*n* = 5) had subthreshold BED, 9% (*n* = 2) had subthreshold BN, 4% (*n* = 1) had purging disorder, and 33% (*n* = 7) could not be classified as a specific OSFED example (i.e., OSFED-other).

#### 3.3.1. Characteristics of OSFED-Other Participants

Of the seven OSFED-other participants, two endorsed regular purging with laxatives in the past three months but did not endorse the overvaluation of shape or weight that would qualify them for purging disorder. Two additional participants exhibited persistent overeating (without loss of control) followed by nonpurging compensatory behaviors (e.g., fasting all day, exercising for five hours), thus missing the criteria for BN. Another participant met all criteria for BED except that the loss of control eating took place over a four-hour period (rather than a two-hour period), resembling grazing behavior. A further participant met all criteria for BED except reporting just two (rather than three) of the five associated binge features, and the last participant met all criteria for BED except that he did not explicitly endorse marked distress in the SCID interview (despite self-reporting clinically significant impairment on the CIA). Notably, all seven participants who received research diagnoses of OSFED-other were also identified as eating disorder cases by clinicians.

Lastly, 5% (*n* = 5) of participants reported consuming at least 25% of their calories after dinner (potentially consistent with the NES variant characterized by “excessive food consumption after the evening meal,” page 354) [1] but were not judged to have either a* DSM-IV* or a* DSM-5* eating disorder by either clinicians or research assessors.

### 3.4. Interrater Reliability of Clinical and Research Diagnoses

Tables 4 and 5 present a cross tabulated comparison of clinical and research diagnoses for* DSM-IV* and* DSM-5*, respectively. The reliability of overarching* DSM-IV* categories (i.e., BN, BED, EDNOS, or no eating disorder) was “substantial” according to Landis and Koch [21] (*κ* = 0.64, 84% agreement). Individual kappas by disorder are listed in Table 6. Under* DSM-5*, the reliability of overarching categories (i.e., BN, BED, OSFED, or no eating disorder) was also “substantial” (*κ* = 0.63, 83% agreement). In contrast, the reliability of* DSM-5* residual categories—including both OSFED examples and the OSFED-other designation—was “poor” (*κ* = 0.15, 31% agreement).

## 4. Discussion

To our knowledge, this is the first study evaluating the point prevalence of* DSM-5* eating disorders in an obesity treatment-seeking sample that did not retrospectively apply* DSM-5* criteria to cases originally diagnosed under* DSM-IV*. This design provided a stronger test of the three main critiques of* DSM-5* revisions, including the potential overdiagnosis of eating disorders among individuals with overweight/obesity; the continued preponderance of EDNOS; and the risk of reduced interrater reliability in real-world settings.

With regard to the first critique, applying* DSM-5* criteria in an obesity treatment setting did not result in a higher than expected prevalence of eating disorders. Indeed, the 32% prevalence of* DSM-5* eating disorders in our weight-loss treatment-seeking sample did not differ significantly from the* DSM-IV* prevalence in the current sample and was comparable to the average* DSM-IV* eating disorder prevalence of 36% reported in a recent review of eating pathology among bariatric surgery candidates [29]. Similarly, the point prevalence of BED in our sample was 9% using both* DSM-IV* and* DSM-5* criteria, which is comparable to previous studies that have found a* DSM-IV* BED point prevalence between 6 and 14% in patients seeking obesity treatment [3]. Also of note, none of our participants met criteria for ARFID, despite recent data indicating that a high proportion of self-identified “picky eaters” are overweight or obese [5]. Importantly, supporting convergent validity,* DSM-5* eating disorder diagnoses were significantly associated with comorbid psychopathology and clinical impairment.

The second main critique of* DSM-5* revisions—that they may not substantially reduce the preponderance of EDNOS [12]—received some support in our study. Not only was OSFED the most common* DSM-5* eating disorder diagnosis, but also “other” was the most common presentation of OSFED. Some of the OSFED-other presentations we observed in the present study have been named and described previously (e.g., “nonpurging compensatory eating disorder,” [30] *n* = 2), whereas others have not. The preponderance of OSFED observed in the present study is similar to findings from previous studies that have applied* DSM-5* criteria retrospectively [13]. Taken together, these data suggest that even a carefully constructed and well thought-out categorical system may be inadequate to fully capture the diversity of eating disorder phenomenology, highlighting the potential benefit of alternative approaches to classification, such as the National Institute of Mental Health's Research Domain Criteria (RDoC) [31].

The third critique of* DSM-5*—that clinicians would have trouble applying the new criteria in the “real world” [9]—was not supported in our study. Rather, results showed moderate to substantial interrater reliability between clinicians and research assessors using* DSM-5* criteria, both for eating disorders overall, as well as for BN, BED, and OSFED individually. This represents no change from* DSM-IV* interrater reliability ratings [14, 17] and is consistent with previous studies of test-retest reliability for* DSM-5* eating disorders [15, 16]. The only exception to this pattern was for residual eating disorders (i.e., individual OSFED examples), which had relatively poor reliability in the present study. Though the small sample size for our OSFED comparisons highlights the need for cautious interpretation, the low level of interrater agreement may well have been due to the lack of operationalized criteria for specific OSFED presentations. In sum, despite the increased complexity of* DSM-5* compared to* DSM-IV* and the historically low reliability between research and clinical diagnoses [14], the overarching* DSM-5* eating disorder categories performed reliably in this naturalistic clinical setting.

An unexpected but interesting observation from the present study was the challenge of distinguishing NES from normative nighttime overeating. Indeed, 6% of participants overall were assigned a diagnosis of NES, as identified through persistent nocturnal eating. In addition, a further 5% who did not receive* DSM-5* eating disorder diagnoses reported consuming more than 25% of their calories after dinner. This latter finding is consistent with a community-based latent class analysis that identified a large category of “nondepressed evening eaters” of variable BMI who reported eating 50% of daily caloric intake after 7 pm, but not after 11 pm, and endorsed few comorbid sleep or mood symptoms [32]. Further research on the optimal method of distinguishing between the evening hyperphagia presentation of NES and normative evening overeating is needed. Redefining NES by late-night eating (post-11 pm) rather than just evening eating (post-7 pm) [32] or requiring additional features such as morning anorexia, insomnia, or the belief that one must eat in order to fall asleep [33] may enhance construct validity and reduce the likelihood of overdiagnosis.

Our findings should be interpreted in light of both strengths and limitations. Regarding strengths, while other studies have compared the prevalence of* DSM-IV* and* DSM-5* eating disorders by retrospectively applying* DSM-5* criteria, this study is the first to prospectively evaluate the validity and reliability of* DSM-5* categories in a weight-loss treatment-seeking sample using a* DSM-5*-based interview. A second strength is that most studies comparing* DSM-IV* and* DSM-5* have focused on samples in which participants are already known to have eating disorders, whereas our use of a weight-loss-treatment sample allowed us to ascertain whether applying* DSM-5 *diagnostic criteria and guidance would misclassify noncases in a nonpsychiatric population. Regarding limitations, the overall sample size was modest, thus limiting power to detect differences in* DSM-IV* versus* DSM-5* prevalence. Further, the low response rate (68%) may have introduced selection bias. Moreover, interrater reliability may have been affected by variables other than* DSM* criteria, such as whether the interview was conducted in-person versus on the telephone, and the small number of clinical (*n* = 2) and research (*n* = 3) assessors. Other limitations relate to the treatment-seeking nature of the sample. For example, bariatric surgery candidates may minimize eating concerns when screening is conducted as part of surgery eligibility, which could have resulted in an underestimation of eating disorder prevalence. Importantly, this limitation is unlikely to have differentially impacted prevalence under* DSM-5 *versus* DSM-IV*, which was our primary research question.

Overall, our findings suggest that the revised* DSM-5* eating disorder criteria may provide greater clinical utility compared to* DSM-IV* and are unlikely to result in a higher prevalence of eating disorders. The lack of clarity in defining NES as well as the high prevalence of OSFED indicate that the criteria may need further refinement in order to most effectively capture eating pathology among individuals with obesity. Thus, the application of dimensional measures (as in RDoC), or frequent updates (e.g.,* DSM-5.1*) that treat* DSM-5* as a “living document” [34], are worthy of serious consideration.

## Figures and Tables

**Table 1:** Demographic characteristics of 100 adults seeking weight-loss treatment.

Sex (%)	
Male	28 (28)
Female	72 (72)

Mean age (SD)	45.8 (12.0) years

Ethnicity (%)∗	
Hispanic/Latino	10 (10)
Not Hispanic/Latino	89 (89)
Race (%)∗	
American Indian/Alaska Native	2 (2)
Black/African American	12 (12)
Asian	2 (2)
Native Hawaiian/other Pacific Islanders	0 (0)
White	81 (81)

Mean BMI (SD)	41.9 (9.1) kg/m^2^

Obesity class (%)	
Overweight (nonobese)	2 (2)
Class I	15 (15)
Class II	29 (29)
Class III	54 (54)

BMI = body mass index.

∗The percentages do not add to 100% because 1 participant did not report ethnicity, and 3 participants did not report race.

**Table 2:** *DSM-IV* versus *DSM-5* eating disorder diagnoses conferred by structured interviews in an outpatient weight-loss seeking sample.

*DSM-IV* research diagnosis (SCID-IV)	*DSM-5* research diagnosis				
	BN	BED	OSFED	No eating disorder	Total
BN	2	0	0	0	2
BED	0	8	1	0	9
EDNOS	0	1	17	0	18
No eating disorder	0	0	3	68	71
Total	**2**	**9**	**21**	**68**	**100**

BN = bulimia nervosa, BED = binge eating disorder, EDNOS = eating disorder not otherwise specified, and OSFED = other specified feeding or eating disorders.

**Table 3:** Psychopathology comparison measured by self-report scales for *DSM-IV *and *DSM-5* between eating disorder and noneating disorder cases.

Research diagnosis	Eating disorder specific psychopathology [mean (SD)]				General psychopathology [mean (SD)]	
	EDE-Q	EDI-3	CIA	BCQ	BDI-II	STAI
*DSM-IV *						
ED	3.35 (0.84)∗	52.52 (14.45)∗	21.13 (9.20)∗	56.41 (25.14)∗∗	16.90 (8.70)∗∗	46.79 (9.73)∗∗
No ED	2.14 (0.99)∗	34.10 (13.09)∗	11.56 (10.57)∗	38.54 (14.81)∗∗	10.80 (9.97)∗∗	39.10 (11.81)∗∗
*DSM-5 *						
ED	3.27 (0.87)∗	50.73 (14.94)∗	19.96 (9.51)∗	55.31 (24.99)∗∗	16.13 (8.71)	46.38 (9.43)∗∗
No ED	2.13 (0.99)∗	34.13 (13.33)∗	11.69 (10.78)∗	38.26 (14.43)∗∗	10.90 (10.15)	38.96 (12.02)∗∗

ED = eating disorder, EDE-Q = Eating Disorder Examination-Questionnaire, EDI-3 = Eating Disorder Inventory eating disorder risk subscale, CIA = Clinical Impairment Assessment, BCQ = Body Checking Questionnaire, BDI-II = Beck Depression Inventory II, STAI = State Trait Anxiety Inventory.

*P < 0.0001 for *t*-test comparing means between ED and no ED, **P < 0.01 for *t*-test comparing means between ED and no ED.

**Table 4:** Eating disorder diagnoses conferred by structured interview for *DSM-IV* (SCID-IV) versus routine clinical interview in an outpatient weight-loss seeking sample.

*DSM-IV* clinician diagnosis	*SCID-IV* research diagnosis				
	BN	BED	EDNOS	No eating disorder	Total
BN	1	0	0	0	1
BED	0	4	3	0	7
EDNOS	1	3	12	4	20
No eating disorder	0	2	3	67	72
Total	**2**	**9**	**18**	**71**	**100**

*κ* = 0.64, 84% agreement; BN = bulimia nervosa, BED = binge eating disorder, and EDNOS = eating disorder not otherwise specified.

**Table 5:** Eating disorder diagnoses conferred by structured interview for *DSM-5* field trial versus routine clinical interview in an outpatient weight-loss seeking sample.

*DSM-5* clinician diagnosis	*DSM-5* research diagnosis								
	BN	BED	OSFED					No eating disorder	Total
			Sub-BN	Sub-BED	PD	NES	Other		
BN	1	0	0	0	0	0	0	0	1
BED	0	4	0	1	0	2	1	0	8
OSFED									
Sub-BED	0	0	1	2	0	0	0	0	3
PD	1	0	0	0	0	0	1	0	2
NES	0	1	1	0	1	1	0	1	5
Other	0	1	0	1	0	0	5	2	9
No eating disorder	0	3	0	1	0	3	0	65	72
Total	**2**	**9**	**2**	**5**	**1**	**6**	**7**	**68**	**100**

*κ* = 0.63, 83% agreement; BN = bulimia nervosa, BED = binge eating disorder, OSFED = other specified feeding or eating disorder, Sub-BN = subthreshold bulimia nervosa, Sub-BED = subthreshold binge eating disorder, PD = purging disorder, and NES = night eating syndrome.

**Table 6:** Interrater reliability between structured interview and routine clinical interview for *DSM-IV* and *DSM-5* eating disorder diagnoses in an outpatient weight-loss seeking sample.

	Kappa	% Agreement	Interpretation (Landis and Koch [21])
*DSM-IV *			
BN	0.66	99%	Substantial
BED	0.46	92%	Moderate
EDNOS	0.55	86%	Moderate
*DSM-5 *			
BN	0.66	99%	Substantial
BED	0.42	91%	Moderate
OSFED			
Sub-BED	0.48	96%	Moderate
PD	−0.01	97%	Poor
NES	0.14	91%	Poor
Other	0.59	94%	Moderate

BN = bulimia nervosa, BED = binge eating disorder, EDNOS = eating disorder not otherwise specified, OSFED = other specified feeding or eating disorder, Sub-BED = subthreshold binge eating disorder, PD = purging disorder, NES = night eating syndrome.
